# Emerging Magnetic Resonance Imaging Techniques and Analysis Methods in Amyotrophic Lateral Sclerosis

**DOI:** 10.3389/fneur.2018.01065

**Published:** 2018-12-04

**Authors:** Andrew W. Barritt, Matt C. Gabel, Mara Cercignani, P. Nigel Leigh

**Affiliations:** ^1^Clinical Imaging Sciences Centre, Brighton and Sussex Medical School, Falmer, United Kingdom; ^2^Hurstwood Park Neurological Centre Haywards Heath, West Sussex, United Kingdom; ^3^Department of Neuroscience, Trafford Centre for Biomedical Research Brighton and Sussex Medical School, Falmer, United Kingdom

**Keywords:** motor neuron disease, MRI—magnetic resonance imaging, event-based model, quantitative magnetization transfer imaging, neurite orientation dispersion and density imaging (NODDI)

## Abstract

Objective markers of disease sensitive to the clinical activity, symptomatic progression, and underlying substrates of neurodegeneration are highly coveted in amyotrophic lateral sclerosis in order to more eloquently stratify the highly heterogeneous phenotype and facilitate the discovery of effective disease modifying treatments for patients. Magnetic resonance imaging (MRI) is a promising, non-invasive biomarker candidate whose acquisition techniques and analysis methods are undergoing constant evolution in the pursuit of parameters which more closely represent biologically-applicable tissue changes. Neurite Orientation Dispersion and Density Imaging (NODDI; a form of diffusion imaging), and quantitative Magnetization Transfer Imaging (qMTi) are two such emerging modalities which have each broadened the understanding of other neurological disorders and have the potential to provide new insights into structural alterations initiated by the disease process in ALS. Furthermore, novel neuroimaging data analysis approaches such as Event-Based Modeling (EBM) may be able to circumvent the requirement for longitudinal scanning as a means to comprehend the dynamic stages of neurodegeneration *in vivo*. Combining these and other innovative imaging protocols with more sophisticated techniques to analyse ever-increasing datasets holds the exciting prospect of transforming understanding of the biological processes and temporal evolution of the ALS syndrome, and can only benefit from multicentre collaboration across the entire ALS research community.

Neuroimaging modalities sensitive to the dynamics and patterns of tissue degeneration in amyotrophic lateral sclerosis (ALS) are required as objective biological markers of disease activity *in vivo*. Standard clinical assessment is usually adequate for diagnosis, however there is a pressing need for non-invasive neuroimaging biomarkers that may differentiate between the various phenotypes within the ALS syndrome, provide more accurate prognostic information, and monitor responses to therapeutic interventions. There is also a need for neuroimaging techniques which have the potential to interrogate the specific mechanisms of neurodegeneration, given that conventional MRI primarily aims to exclude alternative diagnoses (1). As such, it will be important to integrate new modalities of structural and functional imaging (including MRI and PET) with molecular biomarkers of neuronal damage, and indicators of neuroinflammation if the therapeutic impasse for more effective disease treatments is to be broken. Diffusion MRI, particularly diffusion tensor imaging (DTI), has been extensively researched in patients with ALS to infer structural alterations within the brain and spinal cord by virtue of the movement of water molecules induced by magnetic field gradients. Fractional anisotropy (FA) is consistently reduced, often alongside increased mean or radial diffusivity (MD or RD, respectively), within the corticospinal tracts (CSTs) (2–15) and body of the corpus callosum through which pass the fibers connecting hemispheric motor areas (3, 5–8, 10, 12, 16, 17). Indeed, DTI changes are perhaps most reliably encountered within the posterior limb of the internal capsule (18, 19) which forms a common conduit for several descending motor pathways including the CST, cortico-rubro-spinal, and cortico-reticulo-spinal connections (20). Additional areas within the frontal, temporal (11, 21, 22), and parietal areas (11, 23) have shown reduced FA, all of which is consistent with the multisystem motor and extra-motor regions involved clinically and neuropathologically (24–26). Nevertheless, establishing the precise substrate or substrates underlying these changes observed on MRI is not straightforward and may be complimented by novel magnetic resonance imaging techniques and emerging big data analysis methods.

## Neurite Orientation Dispersion and Density Imaging (NODDI)

Diffusion MRI is sensitive to the motion of water molecules at microscopic level. Nevertheless the signal it measures is averaged across volumes of 1–2 mm^3^ (the so-called “voxel”). For this reason, any interpretation of the signal and its origin requires some degree of “modeling.” More than one model has been proposed and each typically incorporates slightly differing mathematical assumptions to interpret and model the signal, thus providing only indirect inferences on anatomical configurations. For instance, DTI assumes that water movement will obey Gaussian properties and is widely accepted to lose consistency when neuronal fibers bend or fan out within a voxel, or where otherwise aligned fiber tracts are crossing each other (5) which is common to areas such as the centrum semiovale and even regions of the foliated corpus callosum (27, 28). Moreover, a reduction in FA signifies changes in both neurite density and orientation dispersion without distinguishing their individual contributions (28, 29). Therefore, variations on the diffusion tensor model have been created in an attempt to address these limitations. One such model is neurite orientation dispersion and density imaging (NODDI).

**Figure 1:**
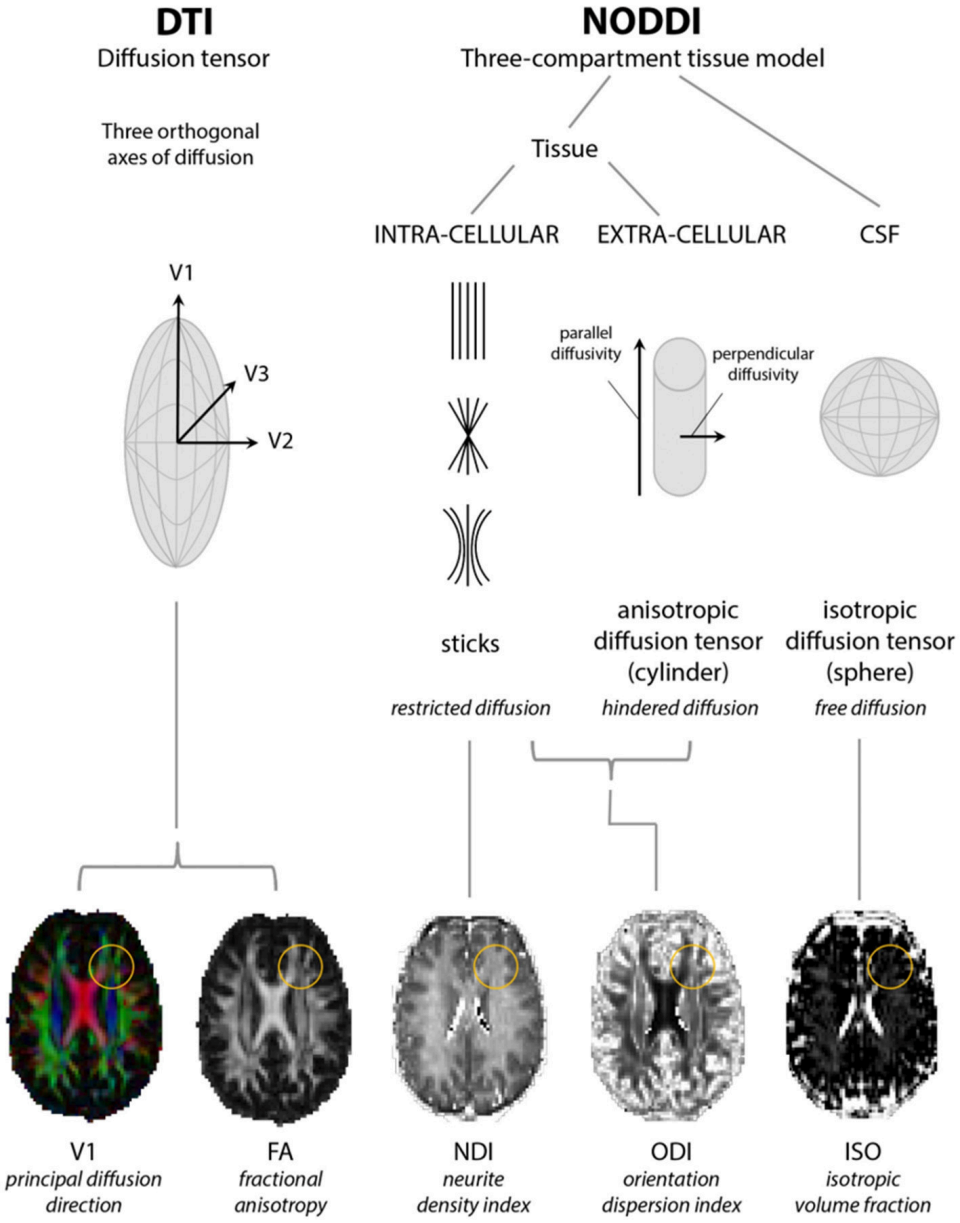
Models of diffusion for neurite orientation dispersion and density imaging (NODDI) and diffusion tensor imaging (DTI). The diffusion tensor model of DTI is based upon three orthogonal axes of diffusion (V1, V2, and V3) yielding radial, axial, and mean diffusivity from which fractional anisotropy (FA) can be estimated. NODDI considers diffusion within three compartments: restricted diffusion in the intracellular compartment, hindered diffusion in the extracellular compartment, and free diffusion in cerebrospinal fluid (CSF), from which parameter maps representing neurite density (NDI), orientation dispersion (ODI), and isotropic fraction (ISO) indices can be estimated. Yellow circles highlight a region where changes in FA can be accompanied by changes in both NDI and ODI. Adapted from Rae et al. (32).

NODDI requires acquisition over a longer time than DTI and compartmentalizes non-Gaussian water diffusion into three geometric spaces encompassing isotropic (or free), hindered anisotropic and restricted anisotropic components. These are known as V_ISO_, V_IC_, and V_EC_ and each broadly correspond to free water/CSF, intra-neurite water (of axons and dendrites), and extra-neurite water (but potentially including glial cells and neuronal somata), respectively (29–31). The NODDI parameters ISO, NDI (neurite density index), and ODI (orientation dispersion index; a marker of the geometric complexity of neurites) can then be derived, the latter two of which are considered to provide a more structurally useful breakdown of single FA values (29) (see Figure 1). NODDI is able to better delineate white from gray matter, in which normal white matter displays higher NDI and lower ODI with the reverse in gray matter (33), and differentiate between different gray matter structures although might be more susceptible to changes in field strength in these areas (31). Compared to DTI, NODDI indices, particularly ODI, have been shown to correlate with histological measures of orientation dispersion in the spinal cord and might also display more inter-subject variability with implications for the sample sizes required for group analyses (33, 34). However, this may not necessarily be an inaccuracy in modeling rather a more accurate depiction of tissue composition (31). In addition, regions which might be expected to demonstrate considerable axon density and higher NDI values might counterintuitively show higher ISO due to the larger diameter axons enabling more freedom of water movement (31, 34).

NODDI has been used to demonstrate tissue alterations associated with normal aging (35–37) and in a range of conditions including focal cortical dysplasia (38), stroke (39), Wilson's disease (40), multiple sclerosis (33), neurofibromatosis type 1 (38, 41), and neurodegenerative diseases. Reduction in NDI and ODI of the contralateral substantia nigra pars compacta has been shown to correlate negatively with clinical severity of Parkinson's disease (42) whereas in pre-manifest Huntington's disease reductions in NDI and ODI are seen in a range of white matter tracts with reduced NDI in the corpus callosum correlating positively with markers of severity (43). In patients with young onset Alzheimer's disease reduction in NDI and ODI is seen corrected for reduced thickness within several relevant cortical areas, with lower NDI values in patients scoring less well on cognitive tests (44), while in a rodent model NODDI indices correlate more consistently than DTI parameters with the burden of tau pathology harbored by the cortex, corpus callosum, and hippocampus (45).

Use of NODDI imaging in ALS has only recently been undertaken. Whole brain analysis in patients with manifest disease has demonstrated a significant NDI reduction throughout the intracranial CSTs up to the subcortical matter of the precentral gyri and across the corpus callosum, with increased ODI in the anterior limb of right internal capsule and increased ISO adjacent to the right lateral ventricle relative to healthy controls (46). NDI within the right corona radiata and precentral subcortical white matter was decreased to a greater extent in those patients with both limb and bulbar involvement compared to limb alone, and longer disease durations correlated with reduced ODI in the precentral gyri, dorsolateral prefrontal cortices, and precuneus. Furthermore, at the statistical threshold used, FA was reduced as expected within the CSTs but less extensively than NDI, and changes were not observed within the corpus callosum, implying NODDI may be more sensitive than DTI. Indeed, combined NODDI and DTI has also been performed in pre-manifest C9orf72 mutation carriers alongside first degree relatives not possessing the pathological repeat expansion (47). The effect size relating to detectable reductions of NDI within 7 of 11 white matter tracts, including the CSTs, is greater than that for DTI metrics (in this case increased axial diffusivity, RD, and MD rather than decreased FA) albeit statistically significant in just two. Therefore, the results appear to corroborate the implication that lowered FA (or increased diffusivity) in the CSTs and corpus callosum results from the loss of axon fibers rather than increased complexity or dispersion within tracts. Longitudinal NODDI scans have not yet been investigated although results from an ancillary imaging study to the Modifying Immune Response and Outcomes in ALS (MIROCALS) trial of low dose Interleukin-2 treatment are awaited.

In any case, neuroimaging techniques are constantly evolving with a raft of acronyms and employing different protocols aiming to reflect the true histological framework of gray and white matter. Although NODDI is considered non-inferior to other MRI modalities of high-angular resolution in this regard (48), it may be that acquisition protocols or MRI data modeling methods undertaken in NODDI, such as spherical (rather than linear) tensor encoding (49) along with tract-based (50), gray matter based (37), and gray matter surface based (51) spatial statistics are further refined in due course to overcome its own recognized limitations.

## Quantitative Magnetization Transfer Imaging (qMTi)

Magnetization transfer imaging, unlike the NODDI model of diffusion MRI, essentially utilizes a “two pool” model in which hydrogen protons are either free or bound to macromolecules (lipids and proteins) within the semisolid tissue. The latter protons do not directly contribute to the MRI signal and are “silent” in diffusion sequences (increased radial diffusivity with DTI is not specific for demyelination) (52), but can be indirectly probed thanks to their interaction with the free protons following off-frequency radiofrequency pulses. The exchange in magnetization between the two compartments allows the state of the semisolid pool (saturated) to affect that of the free protons, resulting in partial saturation and in a decrease of its overall magnetization (53). The magnetization transfer (MT) effect can thereby produce a qualitative magnetization transfer tissue contrast (MTC) image and is already clinically utilized as part of MR angiography and gadolinium-enhanced T1-weighted sequences, for instance. Indeed, MTC T1 images in patients with ALS have shown hyperintensity along the CST (54, 55) and CC (54) in a proportion of cases (and more conspicuously than FLAIR) (55) compared to control subjects which was significantly related to the degree of reduced FA in the same regions and presumed to reflect damage to the white matter tracts, although with no clear association with clinical rating scales or disease duration (54). Acquiring a proton-density image with and without a MT pulse renders it possible to semi-quantify the MT effect and produce a voxel-wise magnetization transfer ratio (MTR) to reflect changes in macromolecular integrity. Accordingly, reduced MTR within the brain has been reported within the CSTs (56), the precentral and other frontal and extramotor gyri (57, 58), in patients with ALS compared to healthy controls, and independently of gray matter atrophy as measured by voxel-base morphometry (57). Significantly reduced average MTR within the spinal cord has also been reported with respect to controls (59–61), accompanied by diminished cord cross-sectional area and average FA (60), and with a longitudinal decline between sequential scans (59). More recent segmentation of the cord into gray and white matter areas, and using a particular adjusted MT protocol called inhomogeneous MT, has demonstrated localized reductions in MTR to the CSTs and dorsal columns in addition to the anterior horns at several non-contiguous cervical levels (62). However, the MTC and MTR are dependent on a range of imaging variables and their biophysical basis is undefined (53).

The development of mathematical models able to describe the MT-weighted signal as a function of the saturating pulses has enabled more biologically applicable parameters to be derived from quantitative magnetization transfer imaging (qMTi), including the macromolecular pool fraction [f; modeled to essentially represent myelin content], forward exchange rate of magnetization transfer [k_f_], and transverse relaxation time of the free pool [T_2_^F^]. Although qMTi is yet to be explored in patients with ALS, studies in multiple sclerosis (MS) have demonstrated reductions in f and k_f_, and increased T_2_^F^ in acute inflammatory lesions with a subsequent return to baseline over several months (63). Compared to healthy controls, normal appearing white matter (NAWM) has reduced f, kf, and MTR (64), and reduced MTR in chronic MS plaques and has been shown to correlate with greater disability (65). Incidentally, reduced MTR in the context of MS is generally considered to be a marker of demyelination, although a small study subdivided NAWM according to distance from a T2 hyper-intense plaque and degree of MTR reduction and found that, whereas at the edge of plaques reduced MTR correlates with reduced myelin content reduced MTR in NAWM may be result from to swollen microglia and, perhaps, axons (66), thus highlighting the uncertainty of its interpretation. MTR in normal appearing gray matter is also reduced in patients with relapsing-remitting MS (67–69) and may also correlate with disability, although variable results are reported (68). Acute increases in kf (but without change in f or T2f) on qMTi have also been induced within the insula in the context of a systemic inflammatory stimulus comprising intramuscular injection of typhoid vaccination and are associated with increased levels of reported fatigue, in addition to a co-localized increase in glucose metabolism measured by FGD-PET (70). Although the mechanisms underlying changes in magnetization transfer parameters are likely to be very different between diseases, it is plausible that qMTi would be sensitive to structural alterations in ALS given the likely role for the immune system in its pathogenesis (71, 72).

## Multimodal MRI

Furthermore, it may be that performing simultaneous qMTi with several other MR neuroimaging sequences, such as diffusion and (resting state) functional MRI, will be most helpful in building a better understanding how both tissue structure and function are affected by the disease process and, ultimately, the difference between certain phenotypes to guide more personalized treatments. Indeed, this is exemplified by the estimations of the myelinated fiber “g-ratio,” the axon diameter divided by the diameter of its ensheathing myelin, which is estimated to ideally be around 0.7 in the central nervous system (73). As diffusion MRI is insensitive to myelin, the combination of intraneurite and isotropic fractions from NODDI and the *f* value from qMTi is required to calculate the g-ratio across the brain. Following adolescence, white matter g-ratio tends to steadily increase with age inferring myelin reduction and knock on effects with respect to the velocity of neuronal conduction (74) and premature increases in the g-ratio are accordingly seen within MS plaques (75, 76). Although ALS is not primarily a demyelinating disease, new insights into the secondary effects of the neurodegenerative process may be revealed with these techniques and correlate with clinical measures.

## Event-Based Modeling

Aside from interpreting the deviations of imaging parameters in terms of current tissue configuration, collecting longitudinal data is, at least conceptually, the most straightforward approach to understanding the temporal evolution of neurodegenerative pathology. However, patient tolerability for repeated MRI acquisition remains challenging in ALS, particularly, due to the rapid accumulation of symptoms and perhaps accounts for the relatively few studies conducted to date (5). Furthermore, it can be argued that participants who are included would be those harboring more slowly-progressing disease, and therefore may not be representative of the majority of patients with ALS.

Given these limitations, alternative methods such as “big data” analysis techniques and new modeling approaches have the potential to greatly increase our understanding of the mechanisms of disease progression. One such approach is the Event-Based Model (EBM) (77–79), a generative probabilistic model originally developed for use in Alzheimer's disease (AD) for which it has been validated in addition to Huntington's disease (80) and recently in ALS using oculomotor data (81). The EBM is designed to extract temporal information from cross-sectional data sets and, unlike traditional models of disease progression, does not rely on *a priori* staging of patients but instead extracts the event ordering directly from the data, thereby minimizing subjective bias.

**Figure 2:**
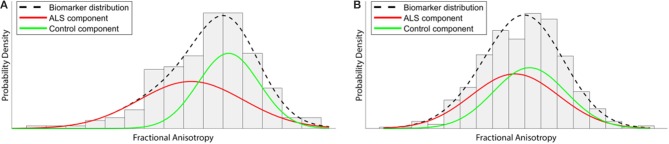
Illustration of how the event-based model (EBM) aims to extract temporal information from a cross-sectional data set. Gaussian distributions of fractional anisotropy (FA) biomarker readings within a tract affected early in the course of the disease, such as the corticospinal tract, would be expected to demonstrate substantial separation between ALS and Control imaging data **(A)**. However, FA from another area affected at later stages demonstrates much less separation between distributions **(B)**. By exploiting and characterizing these differences across all biomarkers, the EBM attempts to order the change from “normal” to “diseased” across the entire disease course.

The EBM defines a disease as a series of “events,” where each event is the change of a biomarker reading from a “healthy” to a “diseased” state. Crucially, biomarker cut-off points are not determined beforehand, but are derived from the data during the modeling process. This not only reduces subjective bias, but also allows for much finer temporal characterization of disease progression than is possible under existing clinically-based staging systems. Healthy control data are used as a fixed reference, and each biomarker is modeled as a mixture of two Gaussian distributions (Figure 2). In order to perform temporal modeling, the EBM assumes that the disease progression is monotonic for individual biomarkers (i.e., the severity of disease burden can only increase). Thus, for biomarkers affected early on in the course of the disease, there will be larger differences between patient and control readings, while biomarkers that are affected late on will have smaller differences between patients and controls. Markov Chain Monte Carlo (MCMC) techniques can then be used to determine the most likely event order across the entire cohort (77).

As with any modeling approach, the EBM has strengths and weaknesses. The ability to extract fine-grained temporal information from cross-sectional data is exceptionally novel and valuable. Use of MCMC techniques also enables the model to quantify the positional variance of individual biomarkers across the cohort, thereby allowing a comparison of their relative importance and variability. In its current form, the EBM reveals aspects of disease progression that are common across the entire cohort (an “average” disease progression). The heterogeneity of ALS means that EBM analyses of stratified subgroups, based on genetic/prognostic factors, are an important future area for investigation.

The accuracy of the EBM output, as with any modeling process, will depend on the quality of the input biomarker data. As a consequence, ALS event-based modeling can require large quantities of data, particularly as individual mean cerebral CST FA values are known to have modest diagnostic power for ALS [found to have a pooled sensitivity and specificity of 0.68 and 0.73, respectively, in a meta-analysis (82)]. Current applications of the EBM to ALS data in progress include analysis of mean FA of white matter (WM) fiber bundles, modeling of patterns of cortical thinning, volumetric changes of brain structures, and oculomotor data. Future areas for development include the application of the EBM to multi-modal ALS biomarker data. Excitingly, the application of the EBM to higher order models of diffusion such as NODDI has the potential to give greater insight into ALS degeneration by simultaneously modeling the changes within ISO, NDI, and ODI parameters.

## Conclusion

Ultimately, all modeling is an attempt to separate meaningful information from randomness. MRI techniques differentially model the signal to derive parameters that plausibly relate to tissue microstructure properties; these parameters can then be modeled further using the EBM to reveal patterns that exist within the data, but which still require human assessment and interpretation (as well as clinical and histological validation). Although the innovative imaging and data analysis techniques presented here constitute a selection of available methods or protocols, their use singly and in combination has the potential to transform our understanding of the biological processes and temporal evolution of ALS, which is likely to benefit further from multicenter collaboration across the entire ALS research community.

## Author Contributions

AWB and MCG performed a review of the literature and drafted the paper. MC and PNL provided specialized expertise and critical appraisal of the article for submission.

### Conflict of Interest Statement

The authors declare that the research was conducted in the absence of any commercial or financial relationships that could be construed as a potential conflict of interest.
